# Heterogeneity of In Vitro Expanded Mesenchymal Stromal Cells and Strategies to Improve Their Therapeutic Actions

**DOI:** 10.3390/pharmaceutics14051112

**Published:** 2022-05-23

**Authors:** Laura Olmedo-Moreno, Yolanda Aguilera, Carmen Baliña-Sánchez, Alejandro Martín-Montalvo, Vivian Capilla-González

**Affiliations:** Department of Regeneration and Cell Therapy, Andalusian Molecular Biology and Regenerative Medicine Centre (CABIMER)-CSIC-US-UPO, 41092 Seville, Spain; laura.olmedo@cabimer.es (L.O.-M.); yolanda.aguilera@cabimer.es (Y.A.); carmen.balina@cabimer.es (C.B.-S.); alejandro.martinmontalvo@cabimer.es (A.M.-M.)

**Keywords:** mesenchymal stem cells, cell therapy, regenerative medicine, cell engineering, drug delivery, preconditioning, scaffolds, secretome, induced pluripotent stem cells

## Abstract

Beneficial properties of mesenchymal stromal cells (MSCs) have prompted their use in preclinical and clinical research. Accumulating evidence has been provided for the therapeutic effects of MSCs in several pathologies, including neurodegenerative diseases, myocardial infarction, skin problems, liver disorders and cancer, among others. Although MSCs are found in multiple tissues, the number of MSCs is low, making in vitro expansion a required step before MSC application. However, culture-expanded MSCs exhibit notable differences in terms of cell morphology, physiology and function, which decisively contribute to MSC heterogeneity. The changes induced in MSCs during in vitro expansion may account for the variability in the results obtained in different MSC-based therapy studies, including those using MSCs as living drug delivery systems. This review dissects the different changes that occur in culture-expanded MSCs and how these modifications alter their therapeutic properties after transplantation. Furthermore, we discuss the current strategies developed to improve the beneficial effects of MSCs for successful clinical implementation, as well as potential therapeutic alternatives.

## 1. Introduction

**Figure 1:**
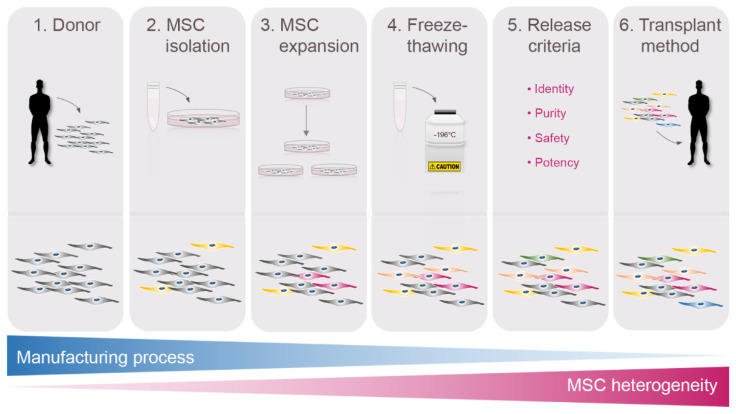
Acquisition of heterogeneity during the manufacturing process of MSCs. MSC heterogeneity increases over the manufacturing process due to multiple factors, such as the particular characteristics of the donor, the isolation method, the conditions of the culture, the freeze-thawing procedure, the release criteria for therapeutic application and the method of administration.

Stem cell-based therapies are expected to benefit patients suffering from a wide spectrum of conditions, mesenchymal stromal cells (MSCs) being the most frequently used cell type. MSCs are multipotent cells that can be obtained from various tissues, including placenta, umbilical cord, amniotic fluid bone marrow, muscle, compact bone, synovial fluid, fat, dental pulp, hair follicles and blood [1]. According to the guidelines of the International Society for Cellular Therapy (ISCT), MSCs must meet three minimal criteria irrespective of their origin [2]. First, MSCs must adhere to plastic surface in standard culture conditions. Second, MSCs must express CD73, CD105 and CD90, while they must be negative for CD45, CD34 and CD14 or CD11b and CD79α or CD19 and HLA-DR. Third, MSCs must differentiate into adipocytes, chondrocytes and osteoblasts under specific in vitro conditions. Over the past decade, MSCs have become the epicenter of regenerative medicine owing to their self-renewal capacity and multilineage differentiation potential, which promote tissue repair and regeneration. The therapeutic actions of MSCs have prompted several preclinical and clinical studies for the treatment of different pathologies, such as neurological disorders, cardiovascular diseases, cartilage lesions or, more recently, the pulmonary complications associated with the severe acute respiratory syndrome coronavirus 2 (SARS-CoV-2) infection [1,3,4,5,6,7]. However, the results obtained in the studies often lack consistency, which have been partially attributed to the functional heterogeneity of MSCs (Figure 1).

The reduced number of MSCs in the different tissue sources implies the need to expand them in vitro to achieve a large-scale production before application. Importantly, the process to culture MSCs is not subject to standardized protocols, which contribute to MSC heterogeneity [8]. The cellular changes initiate early with the method chosen to isolate the cells. Typically, the isolation methods for MSCs are categorized into two main techniques; (1) the enzymatic method, which uses proteolytic enzymes to dissociate tissue into individual cells, and (2) the explant culture method, which is based on the culture of small pieces of the tissue without any digestion step [8]. Similarly, the composition of the culture media can significantly influence the phenotype and function of MSCs. Moreover, cells can be expanded in different plastic surfaces, which have peculiar hydrophobicity characteristics affecting cell growth [9,10]. It is known that the selected culture conditions can induce specific modifications in MSCs, such as changes in their morphology, cell membrane receptor profile and secretome, among other alterations [8,11,12]. Therefore, the lack of common protocols across laboratories enhances the differences in the results obtained when MSC-based therapies are applied. The major goal of this review is to provide a general overview of the modifications occurring in cultured MSCs that may lead to the interlaboratory variability observed when this cell type is used. In addition, we discuss in vitro strategies that may help to obtain more effective MSCs, enabling the improvement of cell-based therapies for a wide range of pathologies, as well as alternative options for the use of MSCs.

## 2. Changes Induced in MSCs during In Vitro Expansion

Several reports have evidenced that MSCs fate is influenced by multiple variables, including the age and pathological condition of the donor [13,14]; tissue source [15]; culture medium composition [16,17]; passage number [13,18,19] or environmental culture conditions, such as oxygen levels, pH or temperature [20,21,22,23]. All these variables may induce cellular and molecular changes through the culture process that affect the therapeutic potential of MSCs. In this section, we analyze the morphological alterations, the variations in the protein expression profile, the discrepancies in the differentiation potential and the physiological perturbations that MSCs undergo during in vitro expansion (Table 1).

### 2.1. Morphological Alterations

The first morphological change observed when culturing MSCs is the shift from spherical to spindle shape when they grow in vitro as adherent cells [38,39]. Furthermore, MSCs are able to adapt to different surface topographies by remodeling their shape as a consequence of cytoplasmic and nuclear shrinkage [38]. On the other hand, MSC morphology can vary in response to medium supplements [40,41], passage number [17] or oxygen conditions [42]. For instance, a study demonstrated that the use of fetal bovine serum (FBS), one of the most widely used supplements in growth media, promotes a flattened shape in MSCs derived from human bone marrow (BM-MSCs), while serum-free media favors a spindle morphology [40]. In addition, BM-MSCs subjected to extensive passage (P6 or higher) become more heterogeneous in terms of morphology, exhibiting differences in granularity and size when examined by flow cytometry [17]. Another factor to consider is the oxygen level in cell cultures. Typically, MSCs reside in hypoxic milieus (1–5% O_2_) when they are in the body [43]. However, during the culture process, MSCs are usually exposed to atmospheric oxygen levels (i.e., 21% oxygen), which affects their intrinsic properties [43]. It has been found that long-term culture of BM-MSCs in normoxic conditions (i.e., 21% oxygen) induce a senescent morphology that is characterized by a flattened and enlarged appearance, accompanied by increased cell volume. In contrast, BM-MSCs cultured under hypoxia retain their spindle-shaped conformation and are smaller in size [35].

### 2.2. Modifications in the Protein Expression Profile

According to the ISCT, MSCs must express CD73, CD105 and CD90 and must not express CD45, CD34, CD14 or CD11b; CD79α or CD19 and HLA-DR. However, there are other markers that can be expressed by MSCs, depending on their tissue origin, donor age or culture condition. For example, STRO-1 is expressed in MSCs derived from bone marrow, adipose tissue, salivary gland, gingiva and synovial fluid, but there is a lack of this antigen in other MSC types, such as those isolated from umbilical cord Wharton’s jelly or peripheral blood [44,45]. The expression of vimentin and stage-specific embryonic antigen (SSEA)-4 has been described in MSCs derived from skin and foreskin [44]. Similarly, several investigations have demonstrated that the marker expression profile of MSCs also changes during in vitro expansion. For example, a report identified that, over cell passages, human gingival MSCs gradually decrease the expression of STRO-1, a marker that defines a MSC purity [45]. This could explain the compromised therapeutic potential of late-passaged MSCs. A separate study showed a variation of surface markers in bovine synovial membrane-derived MSCs throughout the culture period [46]. In particular, they described an initial enhanced expression of CD73 (i.e., passage 1–2) that become reduced after passage 3. Another influencing factor that may lead to modifications in the cell surface protein profiling is the type of culture systems. For instance, a significant decrease of CD146, an endothelial and pericyte marker, has been described in BM-MSCs cultured in a 3D environment, such as 3D microcarrier and spheroid [47,48]. A reduction of CD146 in BM-MSCs may have negative implications, since it has been associated with a lower therapeutic and secretory potency [49].

In addition to cell surface markers, MSCs may differ in the expression of other proteins depending on different factors. For example, a study demonstrated that adipose-derived MSCs (Ad-MSCs) obtained from diabetic patients with critical limb ischemia display an impaired platelet-derived growth factor (PDGF) signaling, compared to Ad-MSCs from healthy individuals [14]. Interestingly, this impaired PDGF signaling was associated with reduced proliferative and migratory capacities. In addition, Ad-MSCs from diabetic patients exhibited the increased levels of tissue factor and plasminogen activator inhibitor type 1, while exhibiting decreased levels of tissue plasminogen activator and d-dimer formation, suggesting a prothrombotic phenotype [14,50]. The expression of pluripotency and differentiation markers is also modified during the culture process. In this regard, the hypoxic culture environment has shown beneficial effects on preserving the stemness properties of MSCs and their multilineage differentiation capacity [35,51,52,53,54,55]. Interestingly, a molecular heterogeneity has been demonstrated between cell profiles of MSCs in vitro and in vivo [56]. A single-cell RNA sequencing analysis found that genes related to telomere length maintenance, including NHP2, CCT6A, RUVBL1 and APEX1, were highly expressed in culture-expanded human umbilical cord MSCs. In contrast, genes related to the epithelial-to-mesenchymal transition, such as BMP2, TGFBR1, VEGFA and CTNNB1, were highly expressed in human umbilical cord tissue cells [56]. This study highlights the need for further studies on MSC biology considering the in vitro and in vivo contexts. In line with this, a recent research work developed an ex vivo culture system based on 3D bone marrow-like scaffolds to characterize hematopoietic stem cells at the molecular level [57]. In particular, they found that cells grown in polydimethylsiloxane scaffolds activated the forkhead box O (FOXO), sterol regulatory element-binding proteins (SREBP) and hypoxia inducible factor 1-alpha (HIF-1α) pathways, which potentiated fatty acids/cholesterol metabolism and reduced mitochondrial respiration, creating an optimized environment for cell expansion.

### 2.3. Discrepancies in the Differentiation Potential

The tissue source is a determinant factor for the differentiation potential of MSCs. A comparative analysis demonstrated that MSCs obtained from bone marrow present a higher osteogenic differentiation potential than MSCs derived from adipose tissue, which are more adipogenic [15]. Similarly, dental pulp stromal cells displayed a greater capacity to differentiate into osteocytes than Ad-MSCs [58]. Another study found that umbilical cord blood MSCs possess a reduced capacity to differentiate into adipocytes, in contrast to BM-MSCs and Ad-MSCs [33,59]. Interestingly, the deficient adipogenic potential of umbilical cord blood MSCs can be rescued by specific induction protocols, such as the addition of calcium to the media [60]. The use of specific protocols to modify the differentiation potential of MSCs is a strategy widely used by researchers. For instance, MSC priming with melatonin was found to increase the osteogenic and chondrogenic differentiation potential of MSCs [61,62,63] while reducing their adipogenic differentiation [64]. The preconditioning of MSCs by hypoxia, cytokines or growth factors are also strategies to improve their differentiation [65]. Donor variations are other modulating factors for the differentiation of MSCs. In line with this, a report demonstrated that BM-MSCs obtained from young donors (0–12 years old) exhibit an increased capacity to differentiate into adipogenic and osteogenic lineages compared to BM-MSCs from adults [13]. Despite age, the health status of the donor may influence the multilineage differentiation potential of MSCs. Several reports have demonstrated that obesity modifies MSC function by affecting the differentiation capacity [29,30]. Importantly, this effect also depends on the tissue source. Thus, MSCs obtained from the bone marrow of obese mice showed a reduced capacity to differentiate into adipocytes, osteoblasts and chondrocytes compared to those from lean mice [30]. In contrast, MSCs derived from the fat of obese mice exhibited increased adipogenic and osteogenic differentiation but decreased chondrogenic potential [30]. Another study indicated that Ad-MSCs derived from diabetic patients are more adipogenic and less osteogenic than those derived from healthy donors [14]. Ad-MSCs isolated from aged individuals have shown angiogenic dysfunction, similar to those obtained from patients with coronary artery disease, due to alterations in the secretion of angiogenic factors, including the vascular endothelial growth factor (VEGF), hepatocyte growth factor and angiogenin [66,67].

### 2.4. Physiological Changes

The maintenance of cellular homeostasis during in vitro expansion is essential to preserve the physiology of MSCs. Cellular homeostasis can be disturbed by changes in the culture conditions, such as oxygen concentration, media supplements or seeding density, thus compromising MSC function. Typically, the oxygen levels applied during the culture process are those that we have in the atmosphere (i.e., 21% oxygen). However, cells in the body are exposed to lower oxygen tensions, ranging from 1% in cartilage and bone marrow to 12% in peripheral blood, which have led scientists to question the optimum oxygen level for cell cultures [43]. This is an important issue because, since, as previously mentioned, culturing MSCs under atmospheric oxygen concentration (21%) may disturb the stemness and differentiation properties of MSCs, affecting cell survival and proliferation [51,52,53,54,55], which is a bottleneck in MSC therapy. Furthermore, MSCs cultured under 21% oxygen levels exhibited increased reactive oxidative species (ROS) accumulation, DNA damage and genetic instability compared to MSCs under hypoxic conditions [68,69]. In line with these observations, a recent study demonstrated that ROS production can be attenuated by reducing the culture temperature (i.e., 35 °C), which enhanced the long-term expansion and adipogenic differentiation of MSCs [70]. In contrast, high temperatures led to an accumulation of ROS and low proliferation [23].

Another factor modulating MSC physiology is the growth surface, since MSCs are characterized by plastic-adherent growth. Consequently, the plastic surface quality is of special relevance for an optimal cell growth. Previous research has demonstrated that the proliferative properties of MSCs vary depending on the brand of culture flasks. In particular, better results were found when cells were grown in Falcon flasks, as compared to other brands (i.e., Greiner, Nunc and Costar), likely due to differences in the surface treatment during the manufacturing process [10]. The stiffness of the culture substrates may also influence several cellular features of MSCs [37,71]. For instance, MSCs cultured on soft substrates (i.e., polydimethylsiloxane—PDMS) presented more relaxed nuclei; the lower maturation of focal adhesions and F-actin assembling; a higher euchromatic content and an augmented expression of pluripotency-related genes (i.e., *NANOG*, *OCT4* and *SOX2*) [37]. Another report demonstrated that cells cultured in different surface topographies exhibited a reduced metabolic activity and slowed the cell cycle progression [38]. In addition, there is evidence that MSCs increase their immunomodulatory effects when they are grown in biomaterials or 3D cultures, as compared to 2D cultures, which may favor their ability to evade immune recognition after transplantation [72].

In addition to the growth surface, there are other specific culture conditions that may affect the immunomodulatory effects of MSCs. For example, the low surface expression level of HLA-I in MSCs, responsible for the immune evasion, can be modified by adding interferon (IFN)-γ to the media [73]. Furthermore, MSCs exhibited a decreased expression of immunosuppressive factors and reduced capacity of inducing regulatory T cells when they were cultured in media supplemented with platelet-poor plasma, as compared to MSCs grown in media with fetal bovine serum [74]. In addition, some studies have demonstrated that passaging and cryopreservation reduce the ability of MSCs to inhibit T-cell proliferation, thus modulating the immune response [75,76,77]. The changes in the immunomodulatory activity of MSCs are particularly relevant when cell therapy is oriented to treat immune-related disorders, such as autoimmune diseases.

## 3. Consequences of the In Vitro MSC Modifications for Their Use in Cell Therapy

MSCs are the most frequently used stem cell type in clinical application due to their therapeutic benefits and their rapid in vitro expansion capacity, among other advantages [1]. However, the changes occurring in cultured MSCs, such as morphological alterations, the different protein expression profiles and the physiological modifications, can limit their use in clinical application. For example, MSCs undergoing extensive size enlargement during in vitro expansion exhibit reduced therapeutic efficacy after systemic administration due to an increased lung entrapment [78,79]. One hour after intravenous administration, most of the infused cells are found in the lungs of mice. Schrepfer and colleagues showed, through fluoresce microspheres of different dimensions, that lung capillaries have an average diameter between 4 and 15 µm in mice. It is suggested that the retention of BM-MSCs in the lungs could be due to the increased size of cultured MSCs, which reach an average diameter of 15–19 µm [79]. The retention of transplanted MSCs in non-target organs results in a lower number of cells in the target tissue, and consequently, the efficacy of the cell therapy diminishes. Furthermore, the use of large MSCs for cell therapy is associated with more frequent adverse events, such as vascular obstructions and stroke, which may compromise the patient’s safety [80,81]. Ge et al. showed that the intracarotid injection of large placental-derived MSCs (average diameter: 29 μm) in rats caused severe vascular obstructions and strokes in comparison with those rats that were injected with smaller placental-derived MSCs (average diameter: 13–17 μm) [80]. Another study with rats demonstrated that the intracarotid administration of MSCs reduced the cerebral blood flow, as opposed to the administration of smaller cells (i.e., glial-restricted precursors) [81].

An additional limitation of expanding MSCs is that senescence processes become activated. Those may be even accelerated under specific culture conditions, inducing MSC functional alterations, such as decreased self-renewal, immunomodulation and differentiation [13,19]. Remarkably, the biological features of in vitro senescent MSCs were similar to those found in MSCs obtained from aged donors, including a decreased proliferation ability and differentiation potential [13,82]. Therefore, the use of long-term expanded MSCs may exhibit restricted therapeutic effects, which can be more pronounced when MSCs are obtained from elderly patients. All these aspects must be taken into consideration when planning clinical trial protocols for MSC-based therapies. The development and implementation of approaches to rejuvenate senescent MSCs would be a promising strategy to enhance the efficacy of cell therapy.

Among senescence-induced alterations, the impaired immunosuppressive function of MSCs is of special relevance for allogeneic cell therapies. When a transplanted cell is recognized by the host immune system as a foreign agent, the mechanisms of immune rejection are activated to eliminate the allograft, hampering the efficacy of cell therapy. Interestingly, MSCs are able to escape immune system recognition and exert immunosuppressive effects through cell-to-cell interactions or paracrine actions [83,84,85], which makes MSCs ideal candidates for immune-related diseases. Even so, this immunomodulatory property can be altered by specific culture conditions, such as cryopreservation, media composition, cell passage or the use of biomaterials [72,74,75,76,77,86]. A recent study demonstrated that human Ad-MSCs exhibit an increased secretion of proinflammatory cytokines after extended culture expansion, while they decrease the secretion of anti-inflammatory cytokines [86]. For this reason, it is crucial to define and validate the potency assays for each specific cellular product before release to support more therapeutically consistent and effective MSCs.

Another important aspect to be considered when planning a cell therapy study is the pathological condition of the donor. As previously mentioned, some diseases may influence the therapeutic properties of MSCs. For instance, reports have demonstrated that Ad-MSCs derived from diabetic patients with critical limb ischemia possess a defective phenotype and exhibit a prothrombotic profile, which was associated with the development of microthrombosis after autologous cell infusion [14,50]. Aging and coronary artery disease have also been associated with the angiogenic dysfunction of MSCs, which may cause an insufficient effectiveness of autologous cell therapy [66,67]. In those cases, where the pathological condition of the donor may restrict the therapeutic properties of MSCs, allogeneic cell therapy would be the preferred option. However, the use of allogeneic cells holds other limitations, the most important being the graft-versus-host disease, which is triggered by the reaction of grafted cells against the host cells. Therefore, further efforts are needed to be oriented towards the development of new strategies to overcome the limitations of both autologous and allogeneic MSC therapy.

## 4. Strategies to Potentiate the Therapeutic Properties of MSCs

The first step to achieve maximum clinical benefits is to optimize the culture conditions for each MSC-based product. It is crucial to define the best culture conditions for the successful in vitro expansion of MSCs, attending to their particular characteristics, including tissue source, age of patient or passage number, among others. In addition, the modification of other culture parameters, such as oxygen level and temperature, can result in an improved therapeutic potential of MSCs. For instance, a study showed that the exposure of MSCs to hypoxia (i.e., 1% oxygen) for 4 and 6 h improved cell migration through the induction of HIF-1α [87]. Modulation of the temperature during the culture process can also promote differences in the proliferation rate of MSCs [23,70], obtaining superior in vitro expansion with a reduced temperature (i.e., 35 °C) [70]. Furthermore, cryopreservation has shown to decrease the T-cell suppressive function of MSCs [75,76]. In this context, the use of freshly or refreshed MSCs has been suggested to improve the efficacy in cell-based therapy. The use of low-passaged MSCs is also recommended to achieve better in vitro expansion [13,19].

**Figure 2:**
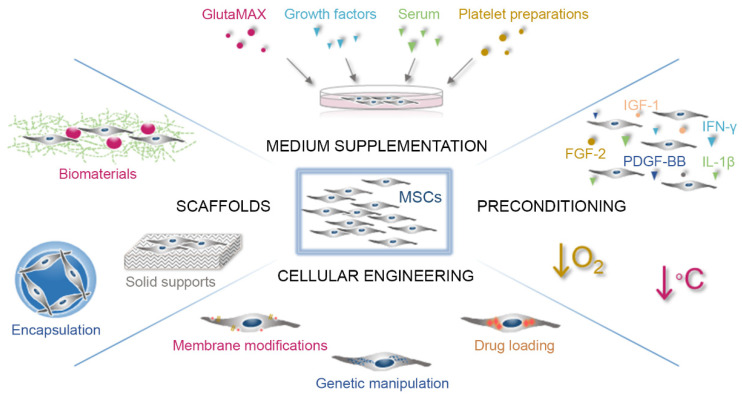
Strategies to potentiate the therapeutic properties of MSCs. Among the most common approaches to improve the MSC therapeutic effects are the use of supplements in the culture media, MSC preconditioning, the use of scaffolds during the seeding or transplantation process and the engineering of MSCs.

In addition to the proper optimization of the culture conditions, multiple strategies have been implemented to improve MSC therapeutic effects. The most common approaches are the use of supplements in the media, MSC preconditioning, the use of scaffolds during the seeding process or the engineering of MSCs (Figure 2).

### 4.1. Use of Medium Supplements

There are multiple medium supplements that have shown beneficial effects to improve the proliferative capacity of MSCs. FBS is the most widely used growth supplement for cell cultures, primarily because of their high levels of growth stimulatory factors and low levels of growth inhibitory factors. The use of FBS has been proven to increase the proliferative capabilities of MSCs in a concentration-dependent manner [88]. However, FBS may contain animal derivatives enhancing the risk of zoonotic transmission and immunological reactions during cell therapy. In this context, human platelet preparations, such as platelet lysate or platelet-rich plasma, have emerged as an alternative to the use of non-human serum, favoring the biological safety of MSC therapies [88,89,90]. Importantly, the clonogenic efficiency and proliferative capacity of MSCs was found to be greater when using 5% human platelet lysate as compared to 10% fetal serum, probably due to the high concentration of natural growth factors contained in platelets [90]. However, both fetal serum and human platelet preparations are complex natural products that may vary from lot to lot, even from a single manufacturer, contributing to the heterogeneity of MSC function and influencing the clinical utility of these cells. Recently, the development of chemically defined media is gaining increasing importance as an alternative to isolate and expand human MSCs for clinical application [91,92]. Although studies have shown multiple improvements in the therapeutic properties of MSCs cultured in media with fully characterized ingredients [93], more efforts should be made to further optimize and standardize the use of the chemical-defined medium.

The use of growth factors is a common strategy to increase the proliferative rate of MSCs. Among the most frequently used growth factors are fibroblast growth factor (FGF), epidermal growth factor (EGF), transforming growth factor beta (TGFβ), PDGF or VEGF [94]. Along with increasing proliferation, these factors present other beneficial effects that may contribute to improve the MSC therapeutic properties. For instance, the supplementation of culture media with FGF was found to reduce apoptosis and senescence [95,96,97]. A study demonstrated that the use of PDGF-BB can reverse the defective phenotype of Ad-MSCs derived from diabetic patients by improving their migration, proliferation and fibrinolytic capacity in vitro [14]. Furthermore, the pretreatment of Ad-MSCs with PDGF-BB promoted the homing of transplanted cells in a mouse model of cutaneous wounds, suggesting an improved migration and survival of MSCs in vivo [14]. Another method to potentiate the therapeutic properties of MSCs is the supplementation of media with GlutaMAX. This supplement increases the proliferation rate of cells, as compared to media supplemented with L-glutamine [10]. This beneficial effect may be due to the fact that GlutaMAX supplement provides a more stable L-glutamine, since it appears as a dipeptide, L-alanine-L-glutamine, that does not spontaneously degrade.

### 4.2. Preconditioning of MSCs

Several studies have used the preconditioning of MSCs to efficiently promote their therapeutic functions. A recent study demonstrated that the melatonin preconditioning of MSCs promoted bone regeneration in an animal calvaria bone defect model by increasing osteogenic differentiation [62]. In a separate study, MSCs pretreated with melatonin exhibited better effects on recovering the liver function than untreated MSCs by enhancing hepatic engraftment after tail vein injection in a mouse liver fibrosis model [98]. The pretreatment of MSCs with mood stabilizers lithium and valproic acid exerted greater benefits on the motor function than non-preconditioned MSCs after intranasal delivery in a mouse model of Huntington’s disease [99]. In particular, this preconditioning enhanced MSC survival post-transplantation and promoted the expression of neurotrophic factors, including FGF-21, FGF-15, neuron-derived neurotrophic factor (NDNF), neurotrophin 3 (NTF3), growth differentiation factor (GDF)-1, insulin-like growth factor (IGF)-1 and bone morphogenetic protein (BMP)-3 [99]. Another research work demonstrated that hypoxia preconditioning effectively promoted the angiogenic capacity of Ad-MSCs obtained from aged donors, increasing the tissue perfusion when they were intramuscularly injected in mice with ischemic hindlimbs [100]. An improved restoration of the blood flow was also observed in the hindlimb ischemia of mice injected with BM-MSCs cultured under hypoxic conditions [101]. In addition, many studies have demonstrated the beneficial effects of pre-stimulating MSCs with cytokines or growth factors [65]. For instance, IFN-γ pre-stimulation increased the immunosuppression properties of MSCs, resulting in diminished mucosal damage after intraperitoneal injection in experimental colitis models [102]. Similar results were found with IL-1β-primed MSCs, which exhibited the enhanced efficacy to ameliorate the pathological aspects of colitis in mice [103]. Another study demonstrated that FGF-2 priming increased the angiogenic capacity of MSCs seeded in tissue constructs and implanted subcutaneously in the back of mice [104]. In particular, this MSC-induced regenerative effect was mediated by the secretion of hepatocyte growth factor (HGF) and VEGF after FGF-2 stimulation. Another study demonstrated that the pretreatment of MSCs with PDGF-BB enhanced engraftment in a cutaneous wound mouse model after tail vein injection [14].

### 4.3. Scaffolds as Structural Supports for MSCs

MSC function depends on multiple conditions, including the environment where they are grown. In order to mimic the natural niche of MSCs, scaffolds have been designed as temporary supports helping to preserve their unique biological behavior and properties. A wide variety of natural and synthetic scaffolds have been successfully used in research [105]. Among natural scaffolds, protein- (e.g., hydrogels, collagen, nanoparticles, fibrin, laminin, etc.) and polysaccharide-based biomaterials (agarose, alginate, hyaluronan, chitosan, cellulose, etc.) are the most commonly used, while synthetic biomaterials are typically made of polymers, such as PDMS [105].

Several reports have used scaffolds in experimental animal models as vehicles to deliver MSCs to the target damaged tissue. A comparative study used different biomaterials to evaluate the retention of transplanted MSCs in a rat model of myocardial infarction [106]. In particular, they used two injectable hydrogels (alginate and chitosan/β-glycerophosphate) and two epicardial patches (alginate and collagen). All these biomaterials increased MSC viability under a hostile milieu and promoted the retention of transplanted cells into the heart [106]. In a recent report, an implantable complex combining collagen and silk fibroin with human umbilical cord MSCs was used to regenerate the cerebral cortex in a canine model of traumatic brain injury (TBI). Animals treated with this combinatorial complex exhibited better brain injury repair than those canines implanted with MSCs alone, as well as a restored gait of hemiplegic limbs after TBI [107]. Another study used polymeric nanofibrils decorated with bovine cartilage-derived decellularized extracellular matrix in combination with MSCs for bone regeneration in a rat osteochondral defect model [108]. The use of this supportive scaffold accelerated chondrogenic differentiation of MSCs, favoring in vivo cartilage regeneration at 12 weeks post-implantation [108].

The use of scaffolds to encapsulate MSCs is another strategy to promote their therapeutic effects, since it confers a protective microenvironment. For instance, MSCs encapsulated in alginate-based scaffolds exhibited improved vascularization in an arteriovenous (AV) loop rat model when examined by histology and microcomputed tomography at 4 weeks post-surgery [109]. Another study showed that MSCs encapsulated within collagen I-hyaluronic acid-based hydrogels display an extended secretion profile of proangiogenic; neuroprotective and immunomodulatory paracrine factors, including VEGF, bFGF and leukemia inhibitory factor (LIF), which favor tissue regeneration [110]. The encapsulation strategy has also been employed to protect transplanted MSCs from the host immune system, which is of special relevance for allogenic cell therapies. Thus, both allogeneic and xenogeneic MSCs that were implanted in immunocompetent rats using a three-dimensional alginate construct persisted for several weeks (i.e., up to 5 weeks post-transplant), while MSCs alone were undetectable after 1 week post-transplant [111]. Importantly, the MSC–alginate constructs preserved their immunomodulatory capacity after long-term in vitro culture by inhibiting T-lymphocyte proliferation and responding to inflammatory stimuli [111]. Therefore, scaffolding can be used as a suitable strategy to potentiate the beneficial effects of MSC-based therapies.

### 4.4. MSC Engineering

An increasing number of researchers are using engineering techniques to refine the therapeutic efficacy of MSCs by promoting their homing toward sites of damage, improving cell survival or enhancing the expression and delivery of therapeutic agent [112,113]. One of the most common techniques to modify MSCs is the genetic manipulation using viral and non-viral vectors to induce the expression of different beneficial factors, such as chemokines and their receptors involved in MSC migration. Numerous reports have engineered MSCs to increase the expression of the C-X-C Motif Chemokine Receptor 4 (CXCR4), which binds to stromal cell-derived factor 1 (SDF1), one of the most potent chemoattractant that is overexpressed at injury sites. Thus, enhanced SDF1/CXCR4 signaling may promote the homing of MSCs into damaged areas to boost tissue regeneration. For example, MSCs transduced to express CXCR4 were injected into the tail veins of rats, 24 h after myocardial infarction. CXCR4-overexpressing MSCs were shown to enhance homing into the infarct region of the myocardium and to improve regeneration when compared to non-transduced MSCs [114]. Similar beneficial effects were found when CXCR4-overexpressing MSCs were transplanted in other injury models, such as the TBI mouse model [115], the acute lung injury rat model [116], the rabbit model of intervertebral disc degeneration [117] or the acute kidney injury mouse model [118].

The genetic manipulation of MSCs has also been used to increase the secretion of pro-regenerative proteins. For instance, genetically modified MSCs to overexpress the antiapoptotic gene Bcl-2 displayed greater survival after injection into rat infarcted myocardium, increasing angiogenesis in the infarct zone [119]. In a preclinical study, MSCs overexpressing VEGF induced more rapid and complete restoration of the blood flow in the ischemic limbs of mice compared to non-transduced MSCs [120], similar to that found with E-selectin-overexpressing MSCs [121]. Genetically modified MSCs can also play an important role in antitumor therapies by directly suppressing cancer progression or by acting as carriers of anticancer payloads [1]. Numerous studies have modified MSCs to overexpress cytokines that potentiate immune responses against cancer, such as interleukin (IL)-12, C-X-C Motif Chemokine Ligand 10 (CXCL10), IFN-β or tumor necrosis factor alpha (TNFα) [113], thus reducing tumor growth and improving survival. The BMP-4 has also been used as tumor-suppressing agent in MSC engineering for cancer therapy. In particular, BMP-4-expressing MSCs efficiently suppressed tumor growth and prolonged the survival of glioma-bearing mice [122,123]. Similar anticancer effects were found when MSCs overexpressing the tumor-suppressor gene Phosphatidylinositol 3,4,5-Trisphosphate 3-Phosphatase (PTEN) were administrated in a xenograft glioma mouse model [124]. However, it must be taken into consideration that the use of MSCs in cancer is still controversial, because there is evidence indicating both protumor and antitumor effects, which hamper the translation to the clinic [1]. The performance of meticulous safety studies would be a required step to anticipate the possible risks of a new cell product during oncological treatment. In line with this, a research group rightly evaluated the possible adverse effects of repeated intranasal MSC administration in mice from early after weaning to adult life [125]. After exhaustive in vivo and ex vivo analyses, they discarded the safety issues of intranasally delivered MSCs in both the short and long term. This report, together with a previous efficacy study, brings new perspectives to face future MSC-based therapies in oncology [125,126].

Despite viral vectors are the most common tool used to genetically modify MSCs with high efficiency, and they clinical application have been limited by the high cost of cell production and safety concerns associated with toxicity, immunogenicity and tumorigenicity [127]. In contrast, nonviral vectors offer a safer option but with a low transfection efficiency [127]. Therefore, the development and optimization of safe and effective gene delivery methods is a major challenge for genetic engineering of MSCs. In this context, emerging reports are using the CRISPR-Cas system to improve the therapeutic potential of stem cells [128]. For example, a study used CRISPR-based technology to improve the therapeutic functions of human MSCs in diabetic wound healing by increasing the secretion of different factors, such as PDGF-BB and VEGFA [129]. More recently, a report used the CRISPR-Cas system to engineer Brain-Derived Neurotrophic Factor (BDNF)-overexpressing MSCs that were applied to effectively treat Rett syndrome in an experimental mouse model [130].

The incorporation of therapeutic cargos into MSCs through cellular internalization or cell surface anchoring is another technique to improve the MSC therapeutic properties [112]. In fact, the use of engineered MSCs as drug delivery systems has emerged as a promising approach for biomedical applications. As MSCs migrate to the target tissue, they deliver their cargo to exert local therapeutic effects, minimizing off-target accumulation and adverse reactions. Among the various types of cargos, drugs, nucleic acids, peptides or nanoparticles are the most frequent in MSC-based therapies. Loading MSCs with therapeutic agents has been particularly used in cancer therapy, where microRNAs (miRNAs) [131,132,133,134], oncolytic viruses [135,136,137] or anticancer drugs [138,139] have shown to be effective against cancer. As an example, a recent study modified the cell surface of MSCs with doxorubicin-encapsulated liposomes and evaluated their therapeutic application in subcutaneous tumor-bearing mice and in a lung metastasis mouse model [140]. Doxorubicin, a common anticancer agent, was efficiently delivered from MSCs and suppressed tumor growth [140]. The field of living drug delivery systems is growing fast and will enable the development of new and more effective forms of therapy for different diseases, improving patient health.

## 5. Alternative Strategies to the Use of Tissue-Derived MSCs

MSCs afford several advantages for cell-based therapy, but their clinical application remains limited due to various factors. First, MSC harvesting usually requires surgical interventions that are, at least, minimally invasive. Second, MSCs need to be expanded before administration to obtain a therapeutic dose, which represents high costs. Third, the application of MSCs may lead to adverse effects, generally associated with thrombotic events, likely due to their large size. Therefore, alternative strategies on the direct use of tissue-derived MSCs have emerged to enable progress in this field.

### 5.1. MSC Secretome

Despite being short-lived, MSCs exert lasting effects after cell delivery through paracrine actions resulting from their secretome, which is rich in soluble proteins, free nucleic acids, lipids and extracellular vesicles, among other elements [141,142]. In fact, several studies have shown the beneficial effects of MSC secretome in the treatment of different diseases [143]. Texeira and colleagues demonstrated that both human umbilical cord mesenchymal progenitors and their secretome efficiently increased the neurogenesis and astrocytic density in the dentate gyrus of the hippocampus after intracranial injection in rats [144]. MSC secretome-mediated therapeutic benefits were also observed in a rat model of Parkinson’s disease. The injection of MSC secretome into the substantia nigra and striatum increased the number of dopaminergic neurons and neuronal terminals, respectively, improving the functional outcomes of the parkinsonian animals [145]. Similar regenerative effects were found after intranasal delivery of the secretome collected from MSCs in an Alzheimer’s disease mouse model [146]. The sustained administration of MSC secretome induced memory recovery in APP/PS1 mice, accompanied by reduced neuroinflammation, a lower density of β-amyloid plaques and augmented neuronal preservation [146]. Protective effects of the MSC secretome were reported after intrahepatic injections in miniature pigs with laparoscopic liver ischemia–reperfusion and partial hepatectomy [147]. The treatment with MSC-conditioned medium restored the fibrosis and hepatic blood parameters in a liver cholestatic fibrosis mouse model by modulating the expression of inflammatory cytokines, such as IL-6, IL-10 and IL-17 [148]. All these reports evidenced that MSC secretome is a potential bioactive pharmaceutical component. Conveniently, the production, handling and storage of secretome is easier and represents a lower cost than MSC manufacturing. Furthermore, the administration of MSC secretome is safer than using live cells, because it bypasses issues related to immune compatibility, tumorigenicity and thrombogenicity.

Extracellular vesicles constitute an important component of the MSC secretome, with a therapeutic capacity for the treatment of several pathologies [149]. Interestingly, the content and secretion of extracellular vesicles vary in response to changes in their environment. Therefore, modifications of the culture conditions may serve as a strategy to obtain more therapeutic effective extracellular vesicles [149]. A recent report demonstrated that the cytokine pretreatment (i.e., TNFα + IFN-γ) of MSCs promoted the anti-inflammatory effects of their extracellular vesicles in primary microglia cultures [150]. Then, using the triple-transgenic mouse model of Alzheimer’s disease (i.e., 3xTg mice), the authors showed that the intranasal administration of extracellular vesicles derived from cytokine pretreated MSCs induced in vivo neuroprotective effects [150]. Another study demonstrated that the exosomes released by HIF-1α-overexpressing MSCs exert increased Jagged 1-mediated angiogenesis in vivo, compared to those exosomes derived from the control MSCs [151]. In addition, MSC-derived extracellular vesicles have been used as vehicles to deliver therapeutic compounds in cancer therapy. MSCs engineered to secrete extracellular vesicles enriched with miRNAs, such as miR-379 and miR-101, exhibited therapeutic effects against breast cancer and metastatic osteosarcoma, respectively [152,153]. Effective anticancer effects were also reported for extracellular vesicles derived from TNF-related apoptosis-inducing ligand (TRAIL)-expressing MSCs by inducing apoptosis in several cancer cell lines and in animal tumor models [154,155]. Taxol-loaded MSC-derived exosomes provide a specific and more efficient tumor-targeting ability than the control exosomes, inducing the cytotoxicity of different human cancer populations, both in vitro and in vivo [156]. Nonetheless, the effectiveness of MSC extracellular vesicles as an anticancer therapy may depend on their tissue of origin. A comparative report demonstrated that extracellular vesicles obtained from bone marrow and umbilical cord MSCs inhibited the in vitro proliferation of the U87 glioma cell line and induced apoptosis, while those obtained from adipose tissue MSCs increased their proliferation and did not affect apoptosis [157]. This study denotes that the heterogeneity of MSC is also present in their derivative products, including extracellular vesicles. To date, therapeutic applications of MSC-derived products remain challenging due to the lack of standardized protocols for its production.

### 5.2. Mesenchymal Stem Cells Derived from Pluripotent Stem Cells

In the past few years, the use of induced pluripotent stem cells (iPSCs) to generate MSCs (iPSC-MSCs) has emerged as a revolutionary option in regenerative medicine. iPSC-MSCs, which closely resemble tissue-derived MSCs, have tropism for injury sites and therapeutic properties. Recent studies have shown beneficial effects of iPSC-MSC and their extracellular vesicles for treating different pathologies, including brain diseases [158,159], respiratory diseases with inflammatory symptoms [160,161], vascular-related pathologies [162,163], immune-mediated conditions [164,165] or cancer [166,167]. Using a hypoxic–ischemic rat model, J. Huang and colleagues demonstrated that the intracranial delivery of iPSC-MSCs reduces the brain pathology and restores motor and cognitive function [158]. In addition, they demonstrated that iPSC-MSCs promote more robust neuroprotective effects than umbilical cord-derived MSCs after brain damage. Interestingly, another study suggested that iPSC-MSCs are safer than BM-MSCs for therapeutic applications in cancer patients, since they do not promote epithelial–mesenchymal transition, invasion or the stemness of cancer cells as BM-MSCs do [167].

Along with potential improved safety and efficacy, the use of iPSC-MSCs has other advantages over tissue-derived MSCs. First, iPSCs can be expanded indefinitely, providing an inexhaustible source of cells for MSC-based therapy and eliminating the bottlenecks in the MSC manufacturing process [168]. Second, the use of iPSCs to generate MSCs bypasses the ethical concerns arising from the use of embryonic cells that involve the destruction of human embryos [169]. Third, iPSC-MSCs are theoretically more homogeneous than tissue-derived MSCs [170], which may contribute to improve the consistency across studies. Finally, iPSC-MSCs obtained from aged individuals acquire a rejuvenation gene signature, which may be associated with a greater proliferation and differentiation capacity than MSCs, as well as higher paracrine activity [171]. Therefore, the use of iPSCs brings about a promising approach for MSC-based therapy.

## 6. Conclusions and Future Perspectives

The use of MSCs in cell-based therapies has shown great promise for the treatment of various diseases, owing to their multiple advantages, including isolation from several sources, large-scale production, tropism for injured sites, and regenerative properties [1]. However, a lack of consistency in preclinical and clinical studies using MSC products highlights the existing functional heterogeneity in these cells. Currently, the challenge for MSC-based therapies is to manufacture homogeneous MSCs to improve the safety and effectiveness of treatments.

MSC heterogeneity depends on multiple factors. Firstly, the age and pathological conditions of the donor, as well as tissue source, may influence MSC function. Accordingly, it is important to know the exact origin of cell batches when planning a cell therapy for success, as well as to know the particular characteristics of these MSCs. In this context, the establishment of cell banks capable of providing well-characterized MSCs would be essential to improve the reproducibility in autologous cell therapies, but there is still much work to be done to ensure the quality and consistency of the final cell products. In addition to cell origin, the different procedures implemented during the cell culture can generate morphological and physiological modifications in MSCs that directly affect their therapeutic potential. Importantly, the lack of a unanimous protocol to culture MSCs aggravates the discrepancies obtained among researchers, which impedes the reproducibility of the results. Therefore, it is of vital importance to improve communication between the different research groups in order to unify the protocols. In this context, the first step to be implemented is the promotion of transparency in scientific publications by providing detailed information regarding the protocols used to culture MSCs. This should include the isolation method; the composition of the media with the references of all reagents; the culture conditions (i.e., oxygen levels, temperature, number of passages, etc.) and the freeze–thawing procedure. In addition, it is important to conduct potency assays related to the intended therapeutic action in order to determine whether a MSC medicinal product is suitable. However, the selection of the appropriate assays relies on the discretion of the researcher, which may contribute to the discrepancies between studies. Definitively, working in a unified protocol will help to advance the basic and clinical research in the field of MSCs and regenerative medicine.

The scientific community has been investigating strategies to enhance the therapeutic properties of MSCs based on the factors influencing their heterogeneity. The main experimental strategies to improve MSC therapeutic properties focus on cell preconditioning, scaffolds and MSC engineering. Selecting the most appropriate strategy will depend on the particular aim of the therapy. For instance, the use of MSCs combined with biomaterials to facilitate their engraftment and survival, thus boosting tissue regeneration, is a good option when performing local cell administration (e.g., intracranial injections or subcutaneous transplant). The priming of MSCs with cytokines is a common strategy to modulate immunosuppression and inflammation [102,103]. Hypoxia preconditioning is the main method to prevent the senescence of MSCs and to improve their angiogenic properties in injured tissues [35,100,101]. Similarly, the use of alternative strategies to the use of tissue-derived MSCs, such as the secretome or iPSC-MSCs, seems to be a convenient option to circumvent some of the barriers of MSC-based therapies, such as the scalable production of medical products.

To date, it is difficult to reach solid conclusions regarding the therapeutic effects of MSC-based therapy in different diseases due to the inherent heterogeneity of MSCs during the manufacturing process. Although many studies have contributed to understanding MSC heterogeneity, further collaborative efforts are necessary to produce consistent final products, driving cell therapy to success.

## Figures and Tables

**Table 1:** Major factors affecting the heterogeneity of MSC during in vitro expansion.

Influencing Factor	Significant Findings	References
Donor variations	- MSCs from young donors are more proliferative than MSCs from adults.	[13]
	- MSCs from cerebellar ataxia patients have lower capacities in terms of proliferation, oxidative stress response, motility, and immunomodulatory functions when compared to healthy individuals	[24]
	- MSCs derived from diabetic individuals exhibit a prothrombotic profile, altered multi-differentiation potential, reduced proliferation, inhibited migration and impaired angiogenic capacity.	[14,25,26]
	- MSCs derived from patients with osteoporotic or osteoarthritis have lower growth rate than control cells.	[27,28]
	- MSCs from obese individual demonstrate decreased differentiation potential, higher expression of HLA-II and CD106, and lower expression of CD29, than cells from non-obese donors.	[29,30]
	- MSCs from patients with myelodysplastic syndrome exhibited reduced clonality and growth, as well as differentiation defects, compared to healthy controls.	[31]
	- Adipogenic and osteogenic differentiation potential of MSCs decreases with age, while chondrogenic potential do not change.	[13]
	- MSCs obtained from Multiple sclerosis patients exhibit senescent appearance in culture and decreased expression of CD105, CD73, CD44 and HLA-A/B/C.	[32]
	- Granularity of MSCs increased with donor age.	[13]
Tissue source	- BM-MSCs possess stronger osteogenic and lower adipogenic differentiation potentials compared to Ad-MSCs.	[15]
	- BM-MSCs express STRO-1, while Ad-MSCs do not.	[33]
Culture medium composition	- The proliferation rate of MSCs grown in xeno-free media is greater than that of MSCs grown in media containing xenogeneic serum.	[16]
	- The use of supplements in the media, such as growth factors (PDGF-BB, TGF-β or bFGF), significantly enhance MSC proliferation.	[14,34]
Incubation conditions	- Alkaline pH (pH > 7.9) negatively affects the MSC osteogenic differentiation and the mineralization process of the extracellular matrix.	[22]
	- High temperature exposure leads to decreased proliferation, cell cycle arrest, and premature senescence of MSCs.	[23]
	- Hypoxia prevent the senescent phenotype of expanded MSCs.	[35]
Growth surface	- MSCs on convex spherical surfaces exhibit more flattened nuclei, compared to cells on concave spherical surfaces.	[36]
	- MSCs migrate faster on concave spherical surfaces, compared to flat and convex spherical surfaces.	[36]
	- MSCs cultured on soft substrates (i.e., polydimethylsiloxane) present more relaxed nuclei, lower maturation of focal adhesions and F-actin assembling, higher euchromatic content, and increased expression of pluripotency-related genes.	[37]
Time on culture	- Prolonged expansion of MSCs gradually impairs DNA damage response and increases chromosomal instability.	[18]
	- Long-term passage of MSCs affects the typical fibroblast-like morphology and decreases proliferation.	[13]
	- Late-passaged MSCs exhibit an altered differentiation capacity, increased proangiogenic potential, and higher expression of the senescence-associated beta-galactosidase (SA-β-gal).	[19]

## Abbreviations

Ad-MSCs	Adipose-derived Mesenchymal Stem Cells
APEX1	Apurinic/Apyrimidinic Endodeoxyribonuclease 1
APP/PS1	Amyloid Precursor Protein/ Presenilin 1
AV	Arteriovenous
BDNF	Brain Derived Neurotrophic Factor
BM-MSCs	Bone Marrow Mesenchymal Stem Cells
BMP	Bone Morphogenetic Protein
Cas	CRISPR-Associated Protein
CCT6A	chaperonin containing TCP1 subunit 6A
CRISPR	Clustered Regularly Interspaced Short Palindromic Repeats
CTNNB1	Catenin beta 1
CXCL10	C-X-C Motif Chemokine Ligand 10
CXCR4	C-X-C Motif Chemokine Receptor 4
EGF	Epidermal Growth Factor
FBS	Fetal Bovine Serum
FGF	Fibroblast Growth Factor
FOXO	Forkhead Box O
GDF	Growth Differentiation Factor
HGF	Hepatocyte Growth Factor
HIF-1⍺	Hypoxia Inducible Factor 1-alpha
IFN	Interferon
IGF	Insulin-like Growth Factor
IL	Interleukin
iPSCs	Induced Pluripotent Stem Cells
ISCT	International Society for Cellular Therapy
LIF	Leukemia Inhibitory Factor
miRNA	MicroRNA
MSCs	Mesenchymal Stromal Cells
NANOG	Nanog Homeobox
NDNF	Neuron-Derived Neurotrophic Factor
NHP2	NHP2 Ribonucleoprotein
NTF3	Neurotrophin 3
OCT4	Octamer-binding transcription factor 4
PDGF	Platelet-derived Growth Factor
PDMS	Polydimethylsiloxane
PTEN	Phosphatidylinositol 3,4,5-Trisphosphate 3-Phosphatase
ROS	Reactive Oxygen Species
RUVBL1	RuvB Like AAA ATPase 1RuvB-Like ATPase 1
SARS-CoV-2	Severe Acute Respiratory Syndrome Coronavirus 2
SDF1	Stromal Cell-derived Factor 1
SOX2	SRY-Box Transcription Factor 2
SREBP	Sterol Regulatory Element Binding Proteins
SSEA	Stage Specific Embryonic Antigen
TBI	Traumatic Brain Injury
TGFBR1	Transforming Growth Factor Beta Receptor 1
TGFβ	Transforming Growth Factor beta
TNF⍺	Tumor Necrosis Factor alpha
TRAIL	TNF-Related Apoptosis Inducing Ligand
VEGF	Vascular Endothelial Growth Factor
VEGFA	Vascular Endothelial Growth Factor A.
